# Formation control for discrete-time multi-agent system with input and output delays under network attacks

**DOI:** 10.1038/s41598-022-12417-9

**Published:** 2022-05-20

**Authors:** Jian Yuan, Hailin Liu, Wenxia Zhang

**Affiliations:** 1 Institute of Oceanographic Instrumentation, Qilu University of Technology (Shandong Academy of Sciences), Shandong Provincial Key Laboratory of Ocean Environment Monitoring Technology, National Engineering and Technological Research Center of Marine Monitoring Equipment, Qingdao, China; Key Laboratory of Ocean Observation Technology, MNR, Tianjin, China; 2 Department of Mechanical and Electrical Engineering, Qingdao City University, Qingdao, China

**Keywords:** Engineering, Mathematics and computing

## Abstract

This paper addresses the mean square consensus-based formation control for multiple agent system (MAS) with input and output delays under network attacks, process noise, and measurement noise in MAS. Firstly, a time delay-free transformation approach for a multi-agent system with input and output delays is presented. The MAS is transformed into a formal delay-free discrete-time system using the delay-free transformation method. Then a state observer based on Kalman filter is presented to estimate the states of agents. A distributed predictive consensus protocol based on the estimated states of each MAS is proposed for the leader-following MAS. The sufficient and necessary conditions of mean square consensus for MAS under DoS attacks are derived. Finally, a numerical example of consensus-based formation control for multi-agent systems is provided to verify the correctness and effectiveness of the proposed consensus control protocol.

## Introduction

Multi-agent systems can solve practical problems with strong robustness, good reliability, and high efficiency. There exist various multi-agent systems in real life, such as multi-robot systems, multiple satellite systems, air vehicle fleet, autonomous underwater vehicle queue, and so on. With the development of sensor network, distributed computing and network communication, network is more and more adopted for information interaction in multiagent systems. Using network technology, multi-agent systems can carry out long-distance data transmission. Due to the openness of the network protocol, network attacks bring inevitably a series of information security problems^1^. By attacking the communication network among multi-agents, the malicious attacks make the communication information unable to transmit normally, which seriously destroys the stability of distributed multi-agent systems. Therefore, increasing attention has been paid to the security consensus of multiagent systems under network attacks^1,2^. The common ways of network attack are denial of service attacks (DoS) and deception attacks, in which denial of service attacks can make the network resources of multiagent systems exhausted. It can temporarily interrupt the networked system or stop the network service, and it can hinder the normal transmission of data and make the data unable to reach the destination agent. At present, the security consensuses of multiagent systems have made some achievements. For example, considering the denial-of-service attacks, the authors studied the distributed consensus control of multiagent systems by using an input-based event triggering method^2^. For the systems with small time-delay, the effect of time-delay was often ignored. However, for the systems with large time delay or in high performance requirements, the effect of time delay cannot be ignored. To solve the time delays of a cohesive formation, Petrillo and Santini, et al. proposed a novel resilient cooperative adaptive strategy that embeds a mitigation mechanism for cyber-attacks which can cope with different malicious behaviors. Using Lyapunov–Krasovkii approach, the effectiveness of the approach was analytically proven when the leading information cannot be falsified with a high-fidelity vehicular network’s emulator PLEXE^3^. Therefore, how to reduce or eliminate the negative effects of time delays has become a hot issue. Wu and He^4^ studied the consensus control problem of networked multi-agent systems with communication time-delay under network attacks. A security consensus algorithm for multiagent systems based on intermediate state values was proposed^5^. Considering the different attacking intensity, Zhang and Feng^6^ introduced the switched system model to model the denial-of-service attacks and studied the robust consensus control of leader–follower multi-agent systems. When the attacking frequency and duration of denial-of-service attacks were less than a certain value, a distributed security control strategy^7^ was proposed to achieve the consensus control of multiagent systems.

In the actual environment, multi-agent systems are often affected by random noise, so the systems often cannot accurately obtain the real states of other agents. If the measured data with noise is directly used, the consensus control method will be difficult to guarantee the system stability of multiagent systems. Existing studies have made significant contributions to address consensus control with noise problems. However, the paper on the consensus of multi-agent systems with noise under network attacking is not enough. The papers^8–10^ showed how to realize the mean square consensus control of multi-agent systems with process noise, measurement noise and communication noise. For the multi-agent systems with process noise and measurement noise, the papers^11,12^ studied the consensus error analysis on the multiagent systems stability. Xia^13^ was concerned with the stochastic stability analysis of networked control systems with random network delay, and then a new control scheme termed networked predictive control was proposed. The control prediction generator was used to provide a set of future control predictions to make the closed-loop system achieve the desired control performance. The Multi-agent systems under external disturbances and network imperfections, i.e., communication delay and random packet dropout, have been considered. The delay-free approach is exploited, and this kind of transformation was adopted in Refs.^14,15^. For the discrete-time systems with time-varying state delay, Gao and Chen^15^ defined a new Lyapunov functions and made use of novel techniques to achieve delay dependence. The merit of the proposed conditions lies in their less conservativeness. The network-delay compensator was adapted to remove the effects of network transmission delay and data dropout. Liu^16^ showed the design and analysis of networked multi-agent predictive control systems via cloud computing, and a cloud predictive control scheme for networked multi-agent systems was proposed to achieve consensus and stability simultaneously and to compensate for network delays actively. The authors concerned the consensus and stability problem of multi-agent control systems via networks with communication delays and data loss, and a networked multi-agent predictive control scheme was proposed to achieve output consensus and compensate for the communication delays and data loss actively^17^. Elahi^18^ considered the third-order dynamics with the rate of change of acceleration under the communication delay and random packet dropout. Using the Lyapunov–Krasovskii function, the sufficient conditions were provided. The consensus error dynamics are asymptotically mean-square consensus stable, and a given disturbance attenuation level is achieved in the presence of both network imperfections and disturbances.

A considerable number of research achievements on consensus control with denial-of-service have emerged. To overcome the disadvantages of continuous time sampling, event-triggered mechanism is proposed. Li and Wang^19^ addresses the mean square consensus problems for leader-following multi-agent systems under denial-of-service attacks, process noise, and measurement noise and a distributed predictive control protocol based on the state estimation information of each agent is proposed for the leader-following multi-agent systems to achieve mean square consensus. Li and Wen^20^ proposed a new adaptive distributed resilient control scheme to resist the influence of the DoS attacks, and it can almost ensure the resilient leader tracking under arbitrary DoS attacks with bounded durations and frequencies. In Ref.^21^ a Markov model approach is proposed to describe the heterogeneous DoS attacks with a particular form of transition probability. Based on the Markov model, an output-feedback observer is designed to estimate the missing sensor data. In Ref.^22^ the authors design a Dynamic Output Feedback Control (DOFC) algorithm to track the given yaw velocity in presence of event-triggering mechanism and DoS attack. Wang^23^ studied the modeling of periodic DoS attacks as Bernoulli distribution, and a novel attack-resilient event-triggered mechanism is put forward to address formation shape problems of the system.

The innovations of this work are as follows. Firstly, considered the time delay in MAS with time delay, a free time-delay transformation for MAS with input and output delays is adopted. Then a states observer based on Kalman filter is presented to estimate the states of multiple agents with random noise. Then a distributed predictive consensus algorithm based on the state estimation information by using Kalman filter and predictive control scheme is proposed, which can effectively achieve the states acquisition of the multi-agent system, so that the leader–follower multi-agent system can achieve better consensus effect, and it further enriches the existing conclusions of prospect theory.

## Problem formation

A discrete-time leader–follower MAS with control and output delays is considered. The dynamic model of agent $i$ is described as

1 $$\begin{gathered} \user2{x}_{i} (k + 1) = A\user2{x}_{i} (k) + B\user2{u}_{i} (k - h_{1} ) + \user2{w}_{i} (k), \hfill \\ \user2{y}_{i} (k) = \left\{ {\begin{array}{*{20}l} {0,k = 0,1, \ldots h_{2} - 1,} \\ {C\user2{x}_{i} (k - h_{2} ) + \user2{v}_{i} (k),k = h_{2} ,h_{2} + 1, \ldots ,} \\ \end{array} } \right. \hfill \\ \user2{x}_{i} (0) = \user2{x}_{{i0}} , \hfill \\ u_{i} (k) = 0,k = 0,1, \ldots ,h_{1} , \hfill \\ \end{gathered}$$

where $k$ is the discrete sampling time, $i \in {\rm N}$, agent 0 is the leader, agent $i$ is the follower $i$, ${\varvec{x}}_{i} (k) \in \Re^{{n_{i} }}$, ${\varvec{y}}_{i} (k) \in \Re^{l}$ and ${\varvec{u}}_{i} (k) \in \Re^{{m_{i} }}$ are the states of $i$, the output states of agent $i$ and control input of agent $i$, respectively. $A \in \Re^{{n_{i} \times n_{i} }}$, $B \in \Re^{{n_{i} \times m_{i} }}$ and $C \in \Re^{{l \times n_{i} }}$ is the parameter matrix of the agent $i$, respectively.$h_{1} > 0$ is the control time delay,$h_{2} > 0$ is the output time delay. ${\varvec{w}}_{i} (k) \in \Re^{{n_{i} }}$ and ${\varvec{v}}_{i} (k) \in \Re^{l}$ is the process noise and measurement noise of the agent $i$, respectively. $Q_{i} (k)$ and $R_{i} (k)$ is the covariance of ${\varvec{w}}_{i} (k) \in \Re^{{n_{i} }}$ and ${\varvec{v}}_{i} (k) \in \Re^{l}$, respectively. The initial state ${\varvec{x}}_{i} (0)$ is uncorrelated with ${\varvec{w}}_{i} (k)$ and ${\varvec{v}}_{i} (k)$.

The solution of (1) can be given by

2 $$\begin{aligned} & {\varvec{x}}_{i} (k) = A^{k} {\varvec{x}}_{i} (0) + \sum\limits_{ii = 0}^{{k - 1 - h_{1} }} {A^{k - ii - 1} } B_{1} {\varvec{u}}_{i} (ii), \\ & {\varvec{y}}_{i} (k) = C_{1} A_{1} {\varvec{x}}_{i} (0) + C_{1} \sum\limits_{ii = 0}^{k - 1 - h} {A^{k - ii - 1} } B_{1} {\varvec{u}}_{i} (ii), \\ \end{aligned}$$

where $$B_{1} = A^{{ - h_{1} }} B$$,$$C_{1} { = }CA_{1}^{{ - h_{2} }}$$,$$h = h_{1} + h_{2}$$.

For the system (Eq. 1), based on the solutions (Eq. 2) a transformation vectors are introduced to convert the time delay system (Eq. 1) into an equivalent delay-free system, the transformed system is described as

3 $$\tilde{\user2{x}}_{i} (k) = {\varvec{x}}_{i} (k) + \sum\limits_{{ii = k - h_{1} }}^{k - 1} {A^{k - ii - 1} } B_{1} {\varvec{u}}_{i} (ii).$$

Meanwhile, a delay-free output transformation vector $\tilde{\user2{y}}_{i} (k)$ is designed as

4 $$\tilde{\user2{y}}_{i} (k) = {\varvec{y}}_{i} (k) + C_{1} \sum\limits_{ii = k - h}^{k - 1} {A^{k - ii - 1} } B_{1} {\varvec{u}}_{i} (ii),$$

where $$\overline{B} = A^{{ - h_{1} }} B$$ and $$\overline{C} = CA^{{ - h_{2} }} .$$

Based on Eqs. (1), (2), (3) and (4), delay system (Eq. 1) can be rewritten as a delay-free form, which is described as

5 $$\begin{gathered} \user2{\tilde{x}}_{i} (k + 1) = A\user2{\tilde{x}}_{i} (k) + \bar{B}\user2{u}_{i} (k) + \user2{w}_{i} (k), \hfill \\ \user2{\tilde{y}}_{i} (k) = C_{1} \left( {\user2{x}_{i} (k) - \sum\limits_{{ii = k - h}}^{{k - 1}} {A_{1}^{{k - ii - 1}} B_{1} u(ii)} } \right) + \user2{v}_{i} (k),k = 0,1,2, \cdots , \hfill \\ \user2{\tilde{x}}_{i} (0) = \user2{\tilde{x}}_{{i0}} \hfill \\ \user2{\tilde{x}}_{{i0}} = \user2{x}_{{\user2{i}0}} . \hfill \\ \end{gathered}$$

It is assumed that the system (Eq. 5) is uniformly completely controllable and uniformly completely observable.

Denial of Service attack is one of the most common network attacks. DoS destroys the information interaction among agents, and it leads that the agent $i$ neighbor data cannot be transmitted to agent $i$, even it seriously affects the consensus of multi-agent systems. The assumption of limited DoS attacker's energy is more realistic. For example, many digital devices usually use battery and when they are used as tools for DoS attacks, the power of DoS attackers is limited. In this paper, we assume that the attacker's energy is limited, so limited continuous attacks can be carried out on the MAS and the upper bound of continuous attack time is $d_{k}$. In addition, the energy constraints of DoS attackers are discussed in Refs.^23,24^. In order to describe the attack probability of DoS, a variable $\alpha_{i} (k)$ subjecting to Bernoulli distribution is defined. When a denial-of-service attack occurs,$\alpha_{i} (k) = 1$, otherwise $\alpha_{i} (k) = 0$. The probability of occurrence or nonoccurrence of denial-of-service attacks are^25^.

$$\left\{ \begin{gathered} \Pr ob\left\{ {\alpha_{i} (k) = 1} \right\} = \alpha \hfill \\ \Pr ob\left\{ {\alpha_{i} (k) = 0} \right\} = 1 - \alpha . \hfill \\ \end{gathered} \right.$$

### Definition 1

If the leader–follower multi-agent systems satisfy $$\lim_{k \to \infty } E\left[ {\tilde{\user2{x}}_{i} (k) - \, \tilde{\user2{x}}_{0} (k)} \right]^{2} = 0,$$$$\forall i \in {\rm N},{\rm N} = \left\{ {1,2, \ldots } \right\}$$.Then the leader–follower multi-agent systems can achieve the mean square consensus.

## Main results

### Observer design

For the leader–follower multi-agent systems (Eq. 5), due to the influence of process noise and measurement noise, the real values of MAS states variables cannot be obtained. So how to deal with the noised information of MAS states effectively and accurately is very important. Based on Kalman filter, an estimation algorithm is proposed to estimate the states of multiple agents effectively, which can reduce the adverse effects of noise on the MAS. The Kalman filter-based observer of leader–follower multi-agent systems (Eq. 5) is designed as follows:

1. Prediction
  $$\begin{gathered} \user2{\hat{\tilde{x}}}_{i} (k + 1\left| k \right.) = A\user2{\hat{\tilde{x}}}_{i} (k\left| k \right.) + \overline{B}{\varvec{u}}_{i} (k), \hfill \\ \user2{\hat{\tilde{y}}}_{i} (k + 1\left| k \right.) = \overline{C}\user2{\hat{\tilde{x}}}_{i} (k + 1\left| k \right.), \hfill \\ P_{i} (k + 1\left| k \right.) = AP_{i} (k\left| k \right.)A^{\text{T}} + Q_{i} (k). \hfill \\ \end{gathered}$$
2. Update
  6 $$\begin{gathered} \user2{\hat{\tilde{x}}}_{i} (k + 1\left| k \right. + 1) = \user2{\hat{\tilde{x}}}_{i} (k + 1\left| k \right.) + K_{i} (k + 1)(\user2{\hat{\tilde{y}}}_{i} (k + 1) - \user2{\hat{\tilde{y}}}_{i} (k + 1\left| k \right.)), \hfill \\ P_{i} (k + 1\left| k \right. + 1) = P_{i} (k + 1\left| k \right.) - K_{i} (k + 1)\overline{C}P_{i} (k + 1\left| k \right.), \hfill \\ K_{i} (k + 1) = P_{i} (k + 1\left| k \right.)\overline{C}^{T} \left[ {\overline{C}P_{i} (k + 1\left| k \right.)\overline{C}^{T} + R_{i} (k + 1)} \right]^{ - 1} , \hfill \\ \end{gathered}$$
  where $K_{i} (k + 1)$ the observer gain, which can ensure the minimum estimation of error covariance $P_{i} (k + 1\left| k \right. + 1)$.

### Controller design

Due to the adverse effects of the process noise, measurement noise and denial of service attacks, how to achieve the consensus of leader–follower multi-agent systems (Eq. 5) has become a very challenging problem. When the denial-of-service attack occurs, the communication data among agents is blocked, the information interaction process cannot be carried out normally, and the information sent by the leader cannot be received by other followers. In this part, based on Kalman filter theory, the states of multi-agent systems with noise are estimated, so that this control scheme can achieve the accurate and effective acquisition of data and provide a fundamental guarantee for the consensus control of leader–follower multiagent systems. Then a distributed predictive control algorithm based on multi-agent states estimation information is proposed by using predictive control theory to ensure the multi-agent systems achieve the consensus in the maximum sustained attacking time.

Taking agent $i$ for an example. When an attack occurs, the agent $i$ uses the state estimation information of its neighbor agent $j$ before time $k$ to predict the states of agent $j$ from the step $k - d_{k} + 1$ to step $k$, where $d_{k} > 0$.The algorithm procedure is as follows:

7 $$\begin{gathered} \user2{\hat{\tilde{x}}}_{j} (k - d_{k} + \tau \left| {k - } \right.d_{k} ) = A\user2{\hat{\tilde{x}}}_{j} (k - d_{k} + \tau - 1\left| {k - } \right.d_{k} ) \hfill \\ \quad + \overline{B}{\varvec{u}}_{j} (k - d_{k} + \tau - 1\left| {k - } \right.d_{k} ), \hfill \\ \end{gathered}$$

8 $$\begin{gathered} \user2{\hat{\tilde{y}}}_{j} (k - d_{k} + \tau - 1\left| {k - } \right.d_{k} ) = \overline{C}\user2{\hat{\tilde{x}}}_{j} (k - d_{k} + \tau \left| {k - } \right.d_{k} ), \hfill \\ \tau = 1,2, \ldots ,d_{k} , \hfill \\ \end{gathered}$$

where $\user2{\hat{\tilde{x}}}_{j} (k)$ is the estimated value of $\tilde{\user2{x}}_{j} (k)$ at time $k$,$\tau$ is time delay, $d_{k}$ is the upper bound of continuous attack time.

To achieve the consensus control of the leader–follower multi-agent system (Eq. 5), a distributed predictive control algorithm based on the estimated states of multi-agents is designed as follows:

9 $$\begin{aligned} {\varvec{u}}_{i} (k) & = G_{i} \sum\limits_{j = 1}^{N} {a_{ij} \left[ \begin{gathered} (1 - \alpha_{i} (k))\user2{\hat{\tilde{x}}}_{j} (k\left| k \right.) \hfill \\ + \, \alpha_{i} (k)\user2{\hat{\tilde{x}}}_{j} (k\left| {k - d_{k} } \right.) - \user2{\hat{\tilde{x}}}_{i} (k\left| k \right.) \hfill \\ \end{gathered} \right]} \\ & \quad + b_{i} G_{i} \left[ \begin{gathered} (1 - \alpha_{i} (k))\user2{\hat{\tilde{x}}}_{0} (k\left| k \right.) \hfill \\ + \, \alpha_{i} (k)\user2{\hat{\tilde{x}}}_{0} (k\left| {k - d_{k} } \right.) \hfill \\ - \user2{\hat{\tilde{x}}}_{i} (k\left| k \right.) + {\varvec{u}}_{0} (k) \hfill \\ \end{gathered} \right], \\ \end{aligned}$$

where $G_{i}$ is the controller gain,$a_{ij}$ is the weight value on the edge between agent $i$ and agent $j$ and $a_{ij} > 0$ if agent $i$ receives the data from agent $j$ else $a_{ij} = 0$.$b_{i} { = }1$ represents the agent $i$ receives the data of the leader else $b_{i} { = 0}{\text{.}}$.

### Consensus analysis

When each agent of the multi-agent systems has process noise and measurement noise, it is difficult to achieve stability for each multi-agent. At the same time, in the process of information interaction among agents of this system, if the system is attacked by denial of service, which makes it more difficult to control the multi-agent system.

#### Theorem 1

*Under the denial-of-service attack, for the multi-agent system* (*Eq.* 5) *with process noise and measurement noise, if matrix* $A_{x}$ *and matrix* $A - K_{i} (k)\overline{C}A$ *satisfy Schur stability theorem, then the multi-agent system* (*Eq.* 5) *can achieve mean square consensus*.

In Theorem 1,$A_{x}$ is described as

$$\begin{aligned} A_{x} & = A_{d} - B_{a} - B_{b} + B_{c} ,A_{d} = diag\left\{ {A,A, \ldots ,A} \right\}, \\ B_{a} & = diag\left\{ {\overline{B}g_{1} G_{1} ,\overline{B}g_{2} G_{2} , \ldots ,\overline{B}g_{N} G_{N} } \right\}, \\ g_{i} & = a_{i1} + a_{i2} + \cdots + a_{iN} ,B_{b} \\ & = diag\left\{ {b_{1} \overline{B}G_{1} ,b_{2} \overline{B}G_{2} , \ldots ,b_{N} BG_{N} } \right\}, \\ B_{c} & = \left[ {\begin{array}{*{20}c} {a_{11} \overline{B}G_{1} } & {a_{12} \overline{B}G_{1} } & \cdots & {a_{1N} \overline{B}G_{1} } \\ {a_{21} \overline{B}G_{2} } & {a_{22} \overline{B}G_{2} } & \cdots & {a_{2N} \overline{B}G_{2} } \\ \vdots & \vdots & \ddots & \vdots \\ {a_{N1} \overline{B}G_{n} } & {a_{N2} \overline{B}G_{n} } & \cdots & {a_{NN} \overline{B}G_{N} } \\ \end{array} } \right]. \\ \end{aligned}$$

#### Proof

For agent $i$, we define the error variable ${\varvec{e}}_{i} (k + 1)$ as

10 $${\varvec{e}}_{i} (k + 1) = \tilde{\user2{x}}_{i} (k + 1) - \user2{\hat{\tilde{x}}}_{i} (k + 1\left| {k + 1} \right.).$$

Combining (2) and (3), we can obtain

11 $$\begin{aligned} {\varvec{e}}_{i} (k + 1) & = \left[ {A - K_{i} (k + 1)\overline{C}A} \right]\left[ {\tilde{\user2{x}}_{i} (k) - \user2{\hat{\tilde{x}}}_{i} (k\left| k \right.)} \right] \\ & \quad - \left[ {I - K_{i} (k + 1)\overline{C}} \right]{\varvec{w}}_{i} (k) - K_{i} (k + 1){\varvec{v}}_{i} (k + 1) \\ & = \left[ {A - K_{i} (k + 1)\overline{C}A} \right]{\varvec{e}}_{i} (k) \\ & \quad - \left[ {I - K_{i} (k + 1)\overline{C}} \right]{\varvec{w}}_{i} (k) - K_{i} (k + 1){\varvec{v}}_{i} (k + 1). \\ \end{aligned}$$

The average value of ${\varvec{e}}_{i} (k + 1)$ is described as

12 $$E\left[ {{\varvec{e}}_{i} (k + 1)} \right] = \left[ {A - K_{i} (k + 1)\overline{C}A} \right]E\left[ {{\varvec{e}}_{i} (k)} \right].$$

Equation (7) can be obtained by iterative calculation

13 $$\begin{aligned} \user2{\hat{\tilde{x}}}_{j} (k\left| {k - } \right.d_{k} ) & = A^{{d_{k} }} \user2{\hat{\tilde{x}}}_{j} (k - d_{k} \left| {k - } \right.d_{k} ) \\ & \quad + \sum\limits_{\tau = 1}^{{d_{k} }} {A^{{d_{k} - \tau }} \overline{B}{\varvec{u}}_{j} (k - d_{k} + \tau - 1)} . \\ \end{aligned}$$

Using the iterative calculation for Eq. (5), we can obtain

14 $$\begin{aligned} \tilde{\user2{x}}_{j} (k) & = A^{{d_{k} }} \tilde{\user2{x}}_{j} (k - d_{k} ) + \sum\limits_{\tau = 1}^{{d_{k} }} {A^{{d_{k} - \tau }} {\varvec{w}}_{j} (k - d_{k} + \tau - 1)} \\ & \quad + \sum\limits_{\tau = 1}^{{d_{k} }} {A^{{d_{k} - \tau }} \overline{B}{\varvec{u}}_{j} (k - d_{k} + \tau - 1)} . \\ \end{aligned}$$

Then Eq. (14) can be rewritten as

15 $$\begin{aligned} & \sum\limits_{\tau = 1}^{{d_{k} }} {A^{{d_{k} - \tau }} \overline{B}{\varvec{u}}_{j} (k - d_{k} + \tau - 1) = } \tilde{\user2{x}}_{j} (k) \\ & \quad - A^{{d_{k} }} \tilde{\user2{x}}_{j} (k - d_{k} ) - \sum\limits_{\tau = 1}^{{d_{k} }} {A^{{d_{k} - \tau }} {\varvec{w}}_{j} (k - d_{k} + \tau - 1)} . \\ \end{aligned}$$

Combining Eqs. (11), (13) and (15), for agent $j$, we can obtain its equation

16 $$\begin{aligned} \user2{\hat{\tilde{x}}}_{j} (k\left| {k - } \right.d_{k} ) & = A^{{d_{k} }} \user2{\hat{\tilde{x}}}_{j} (k - d_{k} \left| {k - } \right.d_{k} ) \\ & \quad + \user2{ \tilde{x}}_{j} (k) - A^{{d_{k} }} \tilde{\user2{x}}_{j} (k - d_{k} ) \\ & \quad - \sum\limits_{\tau = 1}^{{d_{k} }} {A^{{d_{k} - \tau }} {\varvec{w}}_{j} (k - d_{k} + \tau - 1)} = \tilde{\user2{x}}_{j} (k) \\ & \quad - A^{{d_{k} }} \left[ {\tilde{\user2{x}}_{j} (k - d_{k} ) - \user2{\hat{\tilde{x}}}_{j} (k - d_{k} \left| {k - } \right.d_{k} )} \right] \\ & \quad - \sum\limits_{\tau = 1}^{{d_{k} }} {A^{{d_{k} - \tau }} {\varvec{w}}_{j} (k - d_{k} + \tau - 1)} \\ & = \user2{ \tilde{x}}_{j} (k) - A^{{d_{k} }} {\varvec{e}}_{j} (k - d_{k} ) - \sum\limits_{\tau = 1}^{{d_{k} }} {A^{{d_{k} - \tau }} {\varvec{w}}_{j} (k - d_{k} + \tau - 1)} . \\ \end{aligned}$$

In the same way, for agent $0$, we can obtain its equation

17 $$\begin{aligned} \user2{\hat{\tilde{x}}}_{0} (k\left| {k - } \right.d_{k} ) & = \tilde{\user2{x}}_{0} (k) - A^{{d_{k} }} {\varvec{e}}_{0} (k - d_{k} ) \\ & \quad - \sum\limits_{\tau = 1}^{{d_{k} }} {A^{{d_{k} - \tau }} {\varvec{w}}_{0} (k - d_{k} + \tau - 1)} . \\ \end{aligned}$$

Combining Eqs. (16) and (17), the proposed control algorithm (9) can be rewritten as

18 $$\begin{aligned} {\varvec{u}}_{i} (k) & = G_{i} \sum\limits_{j = 1}^{N} {a_{ij} } \left[ {\tilde{\user2{x}}_{j} (k) - \tilde{\user2{x}}_{i} (k)} \right] \\ & \quad + b_{i} G_{i} (\tilde{\user2{x}}_{0} (k) - \tilde{\user2{x}}_{j} (k)) \\ & \quad + G_{i} \sum\limits_{j = 1}^{N} {a_{ij} } \left[ {{\varvec{e}}_{i} (k) - (1 - \alpha_{i} (k)){\varvec{e}}_{j} (k) - \alpha_{i} (k)A^{{d_{k} }} {\varvec{e}}_{j} (k - d_{k} )} \right] \\ & \quad + b_{i} G_{i} \left[ {{\varvec{e}}_{i} (k) - (1 - \alpha_{i} (k)){\varvec{e}}_{0} (k) - \alpha_{i} (k)A^{{d_{k} }} {\varvec{e}}_{0} (k - d_{k} )} \right] \\ & \quad - \alpha_{i} (k)G_{i} \sum\limits_{j = 1}^{N} {a_{ij} } \sum\limits_{\tau = 1}^{{d_{k} }} {A^{{d_{k} - \tau }} {\varvec{w}}_{j} (k - d_{k} + \tau - 1)} \\ & \quad - \alpha_{i} (k)b_{j} G_{i} \sum\limits_{\tau = 1}^{{d_{k} }} {A^{{d_{k} - \tau }} {\varvec{w}}_{0} (k - d_{k} + \tau - 1)} + {\varvec{u}}_{0} (k). \\ \end{aligned}$$

Combining Eqs. (5) and (18), we can obtain

19 $$\begin{aligned} \tilde{\user2{x}}_{i} (k + 1) & = A\tilde{\user2{x}}_{i} (k) + \overline{B}{\varvec{u}}_{i} (k) + {\varvec{w}}_{i} (k) \\ & = A\tilde{\user2{x}}_{i} (k) + \overline{B}G_{i} \sum\limits_{j = 1}^{N} {a_{ij} (\tilde{\user2{x}}_{j} (k) - \tilde{\user2{x}}_{i} (k))} \\ & \quad + \overline{B}G_{i} \sum\limits_{j = 1}^{N} {a_{ij} [{\varvec{e}}_{i} (k) - (1 - \alpha_{i} (k)){\varvec{e}}_{j} (k)} - \alpha_{i} (k)A^{{d_{k} }} {\varvec{e}}_{j} (k - d_{k} )] \\ & \quad + b_{i} \overline{B}G_{i} (\tilde{\user2{x}}_{0} (k) - \tilde{\user2{x}}_{i} (k)) \\ & \quad + b_{i} \overline{B}G_{i} [{\varvec{e}}_{i} (k) - (1 - \alpha_{i} (k)){\varvec{e}}_{0} (k) - \alpha_{i} (k)A^{{d_{k} }} {\varvec{e}}_{j} (k - d_{k} )] \\ & \quad + b_{i} \overline{B}G_{i} (\tilde{\user2{x}}_{0} (k) - \tilde{\user2{x}}_{i} (k)) \\ & \quad + b_{i} BG_{i} [{\varvec{e}}_{i} (k) - (1 - \alpha_{i} (k)){\varvec{e}}_{0} (k) - \alpha_{i} (k)A^{{d_{k} }} {\varvec{e}}_{0} (k - d_{k} )] \\ & \quad + \overline{B}{\varvec{u}}_{0} (k) + {\varvec{w}}_{i} (k) \\ & \quad - \alpha_{i} (k)\overline{B}G_{i} \sum\limits_{j = 1}^{N} {a_{ij} }_{i} \sum\limits_{\tau = 1}^{{d_{k} }} {A^{{d_{k} - \tau }} {\varvec{w}}_{j} (k - d_{k} + \tau - 1)} \\ & \quad - \alpha_{i} (k)b_{i} BG_{i} \sum\limits_{\tau = 1}^{{d_{k} }} {A^{{d_{k} - \tau }} {\varvec{w}}_{0} (k - d_{k} + \tau - 1)} . \\ \end{aligned}$$

For agent $$i$$, we define error variables $$\user2{\overset{\lower0.5em\hbox{$\smash{\scriptscriptstyle\smile}$}}{x} }_{i} (k)$$ as

20 $$\user2{\overset{\lower0.5em\hbox{$\smash{\scriptscriptstyle\smile}$}}{x} }_{i} (k) = {\varvec{x}}_{i} (k) - {\varvec{x}}_{0} (k),$$

Then at time $k + 1$, we can obtain

21 $$\begin{aligned} \user2{\overset{\lower0.5em\hbox{$\smash{\scriptscriptstyle\smile}$}}{x} }_{i} (k + 1) & = \tilde{\user2{x}}_{i} (k + 1) - \tilde{\user2{x}}_{0} (k + 1) \\ & = A\tilde{\user2{x}}_{i} (k) + \overline{B}{\varvec{u}}_{i} (k) + {\varvec{w}}_{i} (k) - A{\varvec{x}}_{0} (k) - \overline{B}{\varvec{u}}_{0} (k) - {\varvec{w}}_{0} (k) \\ & = (A - \overline{B}g_{i} G_{i} - b_{i} \overline{B}G_{i} )\user2{\overset{\lower0.5em\hbox{$\smash{\scriptscriptstyle\smile}$}}{x} }_{i} (k) \\ & \quad + \overline{B}G_{i} \sum\limits_{j = 1}^{N} {a_{ij} \user2{\overset{\lower0.5em\hbox{$\smash{\scriptscriptstyle\smile}$}}{x} }_{j} (k) + (Bg_{i} F_{i} + b_{i} \overline{B}G_{i} ){\varvec{e}}_{i} (k)} \\ & \quad - (1 - \alpha_{i} (k))\overline{B}G_{i} \sum\limits_{j = 1}^{N} {a_{ij} {\varvec{e}}_{j} (k)} \\ & \quad - \alpha_{i} (k)\overline{B}G_{i} \sum\limits_{j = 1}^{N} {a_{ij} A^{{d_{k} }} {\varvec{e}}_{j} (k - d_{k} )} - (1 - \alpha_{i} (k))b_{j} \overline{B}G_{i} {\varvec{e}}_{0} (k) \\ & \quad - \alpha_{i} (k)b_{j} \overline{B}G_{i} A^{{d_{k} }} {\varvec{e}}_{0} (k - d_{k} ) + {\varvec{w}}_{i} (k) - {\varvec{w}}_{0} (k) \\ & \quad - \alpha_{i} (k)\overline{B}G_{i} \sum\limits_{j = 1}^{N} {a_{ij} \sum\limits_{\tau = 1}^{{d_{k} }} {A^{{d_{k} - \tau }} } {\varvec{w}}_{j} (k - d_{k} + \tau - 1)} \\ & \quad - \alpha_{i} (k)b_{j} \overline{B}G_{i} \sum\limits_{\tau = 1}^{{d_{k} }} {A^{{d_{k} - \tau }} {\varvec{w}}_{0} (k - d_{k} + \tau - 1)} . \\ \end{aligned}$$

The average value of Eq. (21) can be described as

22 $$E\left[ {X(k + 1)} \right] = A_{x} E\left[ {X(k)} \right] + E\left[ {M(k)} \right],$$

where

$$E\left[ {X(k)} \right] = \left[ {\begin{array}{*{20}c} {E\left[ {\user2{\overset{\lower0.5em\hbox{$\smash{\scriptscriptstyle\smile}$}}{x} }_{1}^{T} (k)} \right]} & {E\left[ {\user2{\overset{\lower0.5em\hbox{$\smash{\scriptscriptstyle\smile}$}}{x} }_{2}^{T} (k)} \right]} & \cdots & {E\left[ {\user2{\overset{\lower0.5em\hbox{$\smash{\scriptscriptstyle\smile}$}}{x} }_{N}^{T} (k)} \right]} \\ \end{array} } \right]^{T} ,$$

$$E\left[ {M(k)} \right] = \left[ {\begin{array}{*{20}c} {E\left[ {{\varvec{m}}_{1}^{T} (k)} \right]} & {E\left[ {{\varvec{m}}_{2}^{T} (k)} \right]} & \cdots & {E\left[ {{\varvec{m}}_{N}^{T} (k)} \right]} \\ \end{array} } \right]^{T} ,$$

$$\begin{aligned} E\left[ {{\varvec{m}}_{i} (k)} \right] & = (\overline{B}g_{i} G_{i} + b_{i} \overline{B}G_{i} )E\left[ {{\varvec{e}}_{j} (k)} \right] \\ & \quad - (1 - \alpha )\overline{B}G_{i} \sum\limits_{j = 1}^{N} {a_{ij} E\left[ {{\varvec{e}}_{j} (k)} \right]} \\ & \quad - \alpha \overline{B}G_{i} \sum\limits_{j = 1}^{N} {a_{ij} A^{{d_{k} }} E\left[ {{\varvec{e}}_{j} (k - d_{k} )} \right]} \\ & \quad - (1 - \alpha )b_{i} \overline{B}G_{i} E\left[ {{\varvec{e}}_{0} (k)} \right] - \alpha b_{i} \overline{B}G_{i} A^{{d_{k} }} E\left[ {{\varvec{e}}_{0} (k - d_{k} )} \right]. \\ \end{aligned}$$

From Eq. (12), we can obtain

23 $$E\left[ {{\varvec{\varepsilon}}(k + 1)} \right] = \overline{B}_{e} E\left[ {{\varvec{\varepsilon}}(k)} \right],$$

where

$$\begin{gathered} E\left[ {\user2{\varepsilon }(k)} \right] = \left[ {\begin{array}{*{20}c} {E\left[ {\user2{e}_{0} (k)} \right]^{T} ,} & {E\left[ {\user2{e}_{1} (k)} \right]^{T} ,} & \cdots & {,E\left[ {\user2{e}_{N} (k)} \right]^{T} } \\ \end{array} } \right., \hfill \\ \left. {\begin{array}{*{20}c} {E\left[ {\user2{e}_{0} (k - d_{k} )} \right]^{T} ,} & {E\left[ {\user2{e}_{1} (k - d_{k} )} \right]^{T} ,} & \cdots & {E\left[ {\user2{e}_{N} (k - d_{k} )} \right]^{T} } \\ \end{array} } \right]^{T} \hfill \\ \end{gathered}$$

$$\begin{gathered} \overline{B}_{e} = diag\left\{ {\begin{array}{*{20}c} {A - K_{0} (k)\overline{C}A,} & {A - K_{1} (k)\overline{C}A,} & { \cdots ,} & {A - K_{N} (k)\overline{C}A,} \\ \end{array} } \right. \hfill \\ \left. {\begin{array}{*{20}c} {A - K_{0} (k)\overline{C}A,} & {A - K_{1} (k)\overline{C}A,} & { \cdots ,} & {A - K_{N} (k)\overline{C}A} \\ \end{array} } \right\}. \hfill \\ \end{gathered}$$

Combining Eqs. (22) and (23), the error equation can be rewritten as

24 $$\left[ {\begin{array}{*{20}c} {E\left[ {X(k + 1)} \right]} \\ {E\left[ {{\varvec{\varepsilon}}(k + `1)} \right]} \\ \end{array} } \right] = \left[ {\begin{array}{*{20}c} {A_{x} } & {\overline{B}_{e} } \\ 0 & {\overline{B}_{e} } \\ \end{array} } \right]\left[ {\begin{array}{*{20}c} {E\left[ {X(k)} \right]} \\ {E\left[ {{\varvec{\varepsilon}}(k)} \right]} \\ \end{array} } \right],$$

where

$$\overline{B}_{e} E\left[ {{\varvec{\varepsilon}}(k)} \right] = E\left[ {M(k)} \right].$$

Obviously, when the error Eq. (24) is asymptotically stable, the leader–follower multiagent system (Eq. 5) can achieve mean square consensus control. According to the Schur stability theorem, if and only if the matrix $A_{x}$ and $A - K_{i} (k)\overline{C}A$ satisfy Schur stability theorem, there are

$$\mathop {\lim }\limits_{x \to \infty } E\left\| {\tilde{\user2{x}}_{i} (k)} \right.\left. { - \tilde{\user2{x}}_{0} (k)} \right\| = 0,\forall i \in N.$$

Furthermore, when

$$\mathop {\lim }\limits_{x \to \infty } E\left\| {\tilde{\user2{x}}_{i} (k)} \right.\left. { - \tilde{\user2{x}}_{0} (k)} \right\|^{2} = 0,\forall i \in N,$$

the leader–follower multi-agent system (Eq. 5) can achieve mean square consensus, and then the system (Eq. 1) can achieve the mean square consensus.

## Simulation analysis

**Figure 1:**
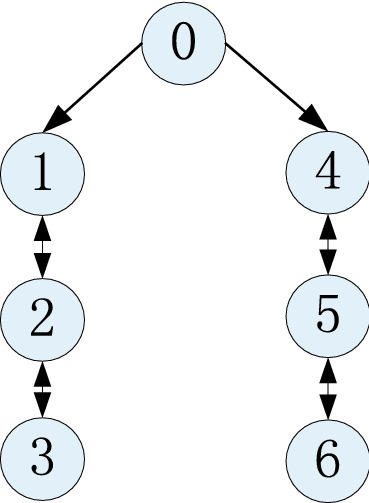
The MAS’ communication topology.

Consider a multi-agent system composed of one leader and six followers. The MAS’ topology is shown in Fig. 1, in which agent 0 is the leader, the others are followers, and agent 1 and agent 4 can receive the leader's information.

The matrix ${\mathbf{H}} = diag\left\{ {1,0,0,1,0,0} \right\}$ and the related parameters and matrix are

$$A = \left[ {\begin{array}{*{20}c} {1.2} & {0.25} \\ { - 0.2} & 1 \\ \end{array} } \right],B = \left[ {\begin{array}{*{20}c} 0 \\ 1 \\ \end{array} } \right],C = \left[ {\begin{array}{*{20}c} 1 \\ 0 \\ \end{array} } \right]^{T} ,D = \left[ {\begin{array}{*{20}c} {0.8} & 0 \\ 0 & {0.9} \\ \end{array} } \right],$$

$$\begin{aligned} & Q_{i} (k) = diag\left\{ {\begin{array}{*{20}c} {10^{ - 3} ,} & {10^{ - 3} } \\ \end{array} } \right\},R_{i} (k) = 2 \times 10^{ - 3} , \\ & P_{i} (\left. 0 \right|0) = diag\left\{ {\begin{array}{*{20}c} {10^{ - 3} ,} & {10^{ - 3} } \\ \end{array} } \right\},i = 0,1,2,3. \\ \end{aligned}$$

### Example 1

The probability of denial-of-service attack $\alpha = 0.6$. The upper bound of continuous attack time $d_{k} = 2$ and the initial states of all agents are.

$${\varvec{x}}_{0} (0) = \left[ {\begin{array}{*{20}c} 1 & 5 \\ \end{array} } \right]^{T} ,$$ $${\varvec{x}}_{1} (0) = \left[ {\begin{array}{*{20}c} {1.5} & 1 \\ \end{array} } \right]^{T} ,$$ $${\varvec{x}}_{2} (0) = \left[ {\begin{array}{*{20}c} {2.5} & 2 \\ \end{array} } \right]^{T} ,$$ $${\varvec{x}}_{3} (0) = \left[ {\begin{array}{*{20}c} {3.8} & 4 \\ \end{array} } \right]^{T} ,$$ $${\varvec{x}}_{4} (0) = \left[ {\begin{array}{*{20}c} {2.1} & {3.5} \\ \end{array} } \right]^{T} ,$$ $${\varvec{x}}_{5} (0) = \left[ {\begin{array}{*{20}c} {1.8} & {2.6} \\ \end{array} } \right]^{T} ,$$ $${\varvec{x}}_{6} (0) = \left[ {\begin{array}{*{20}c} {1.5} & 3 \\ \end{array} } \right]^{T} .$$

The leader's control input ${\varvec{u}}_{0} (k) = 0.8.$ Choose a set of system gain

$$G_{1} (0) = \left[ {\begin{array}{*{20}c} { - 0.2} & {0.4} \\ \end{array} } \right],$$$$G_{2} (0) = \left[ {\begin{array}{*{20}c} { - 0.3} & {0.04} \\ \end{array} } \right],$$$$G_{3} (0) = \left[ {\begin{array}{*{20}c} { - 0.35} & {0.14} \\ \end{array} } \right],$$$$G_{4} (0) = \left[ {\begin{array}{*{20}c} { - 0.23} & {0.25} \\ \end{array} } \right],$$ $$G_{5} (0) = \left[ {\begin{array}{*{20}c} {0.3} & {0.44} \\ \end{array} } \right],$$ $$G_{6} (0) = \left[ {\begin{array}{*{20}c} { - 0.43} & {0.04} \\ \end{array} } \right].$$

**Figure 2:**
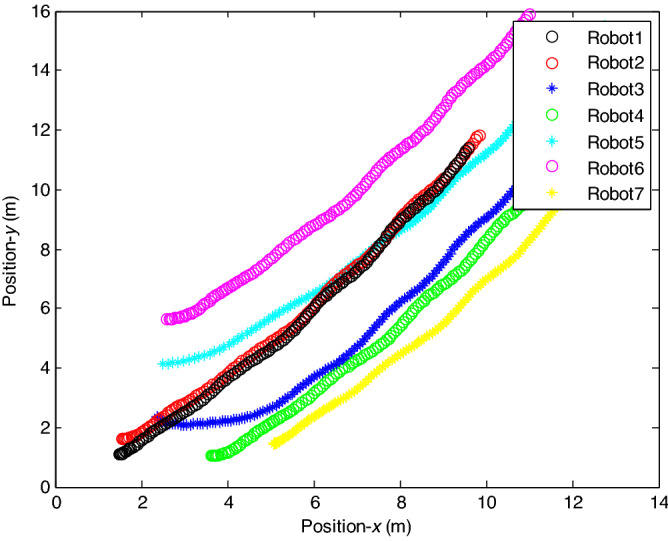
Consensus control of seven agents.

**Figure 3:**
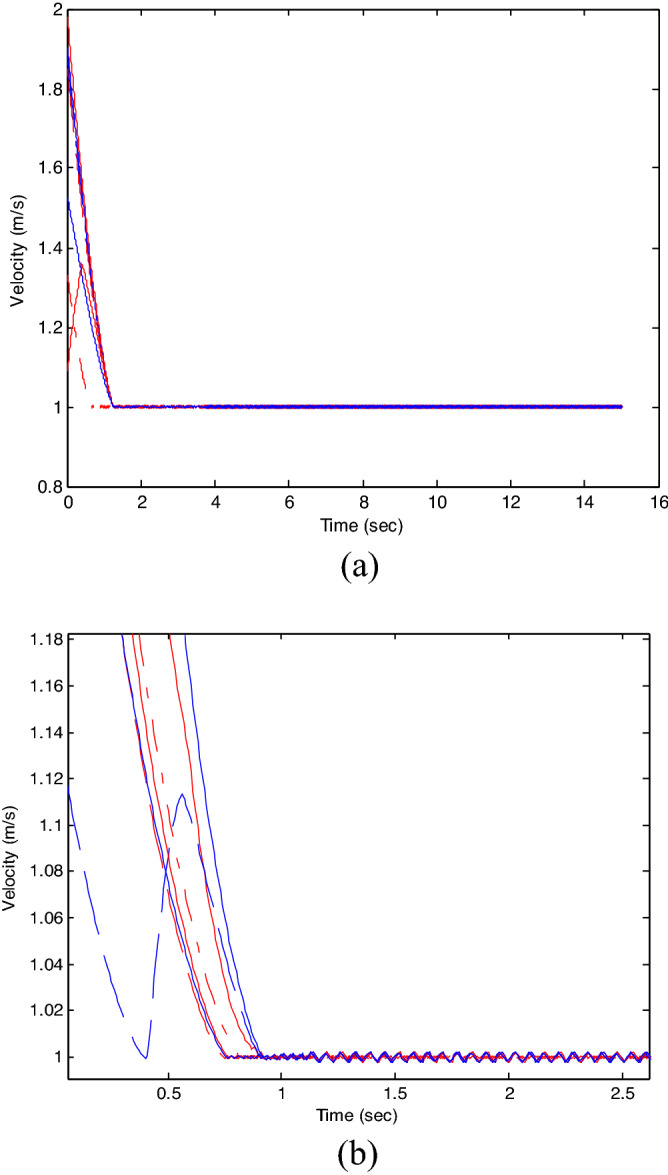
Consensus control on velocity of seven agents (**b**) local magnification of figure (**a**).

**Figure 4:**
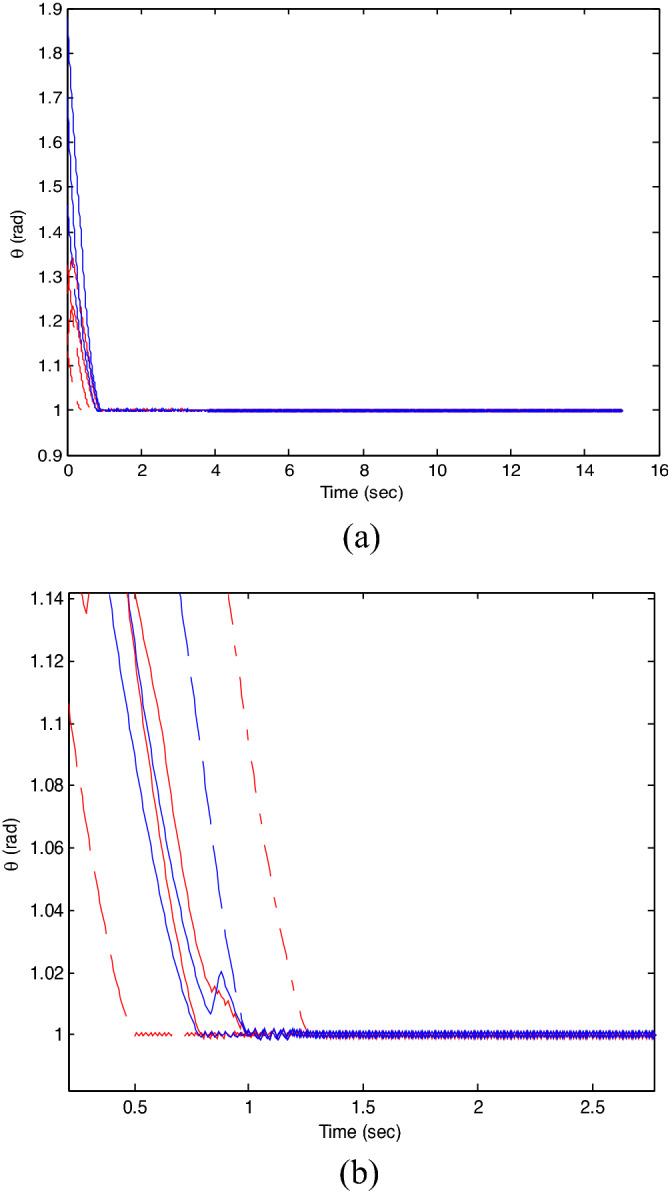
Consensus control on heading angle of seven agents (**b**) local magnification of figure (**a**).

The control time delay $h_{1} = 2$, and the output time delay $h_{2} = 1$.Note that the matrices $A_{x}$ and $A - K_{i} (k)CA(\forall i \in N)$ matrices are of Schur stability. Figure 2 shows the consensus control motion simulation of seven agents at constant velocity and heading angle, Fig. 3 shows the speed consensus control and Fig. 4 shows the heading angle consensus control. The consensus control of seven agents is well realized. The simulation results show that the designed state estimator can effectively estimate the states of multiple agents and compensate the lost states. Although the network attacks are frequent, the use of system state estimator can reduce the impact of time delay from network attaches. The state estimating scheme can make the states of multiple agents’ rapidly convergence to the predefined value.

### Example 2

**Figure 5:**
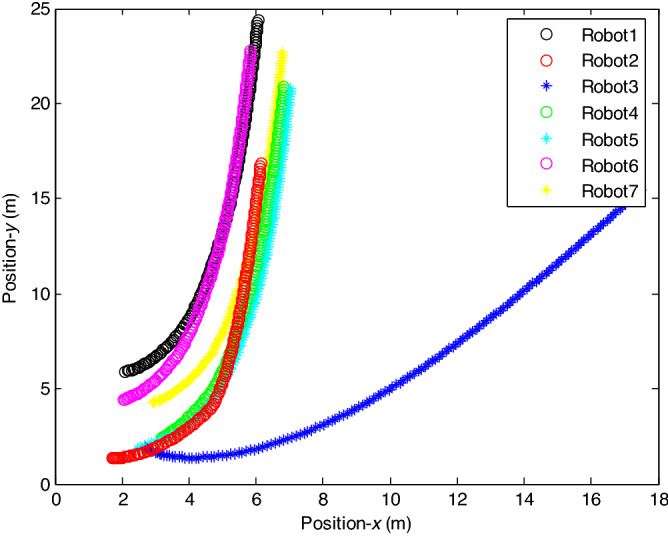
Consensus control of seven agents.

**Figure 6:**
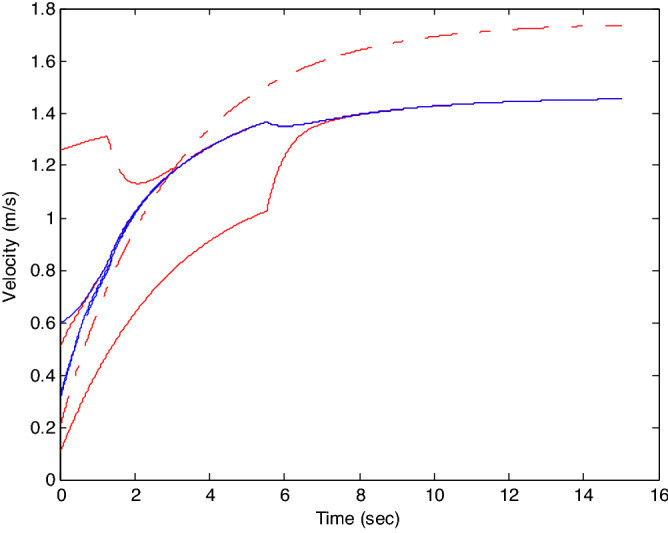
Consensus control on velocity of seven agents with attacks at initial time.

**Figure 7:**
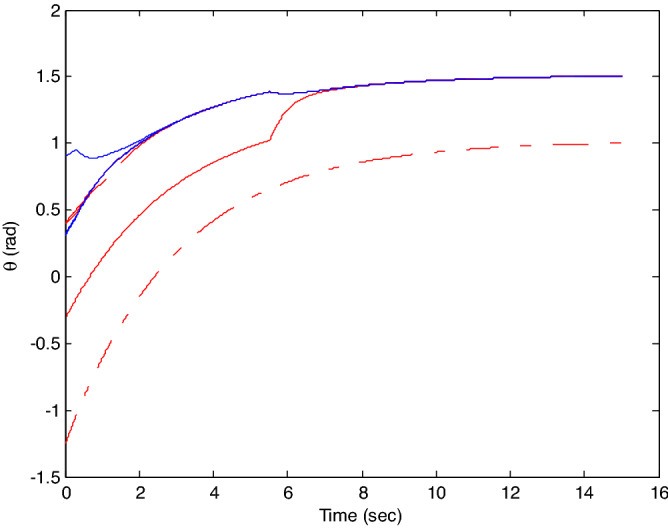
Consensus control on heading angle of seven agents with attacks at initial time.

**Figure 8:**
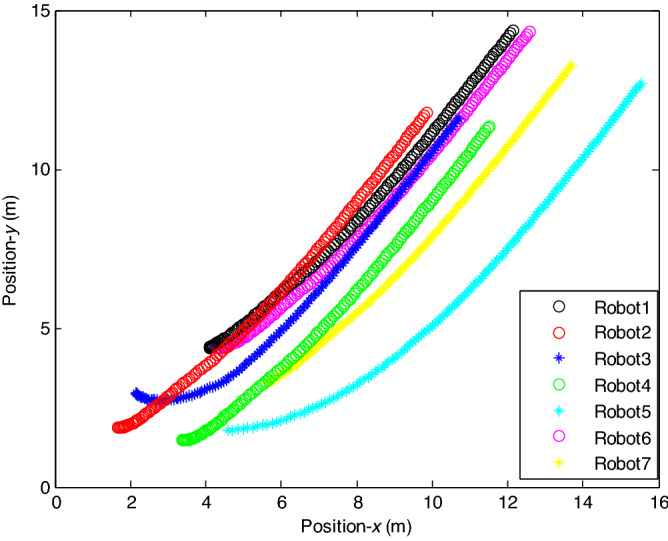
Consensus control of seven agents.

**Figure 9:**
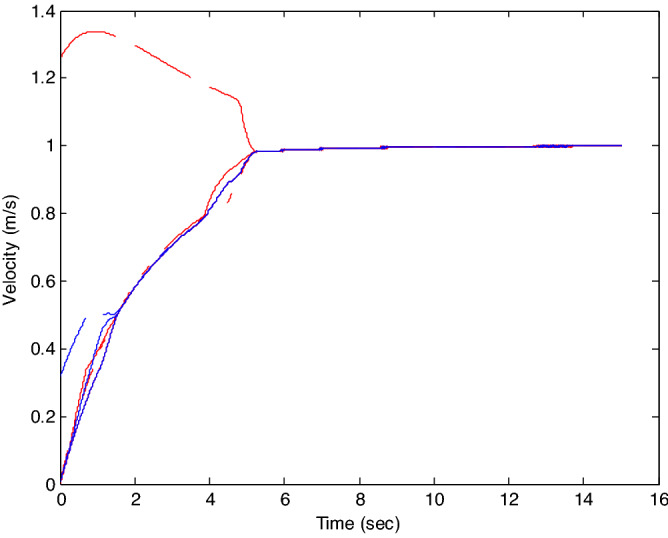
Consensus control on velocity of seven agents with attacks at one second interval.

**Figure 10:**
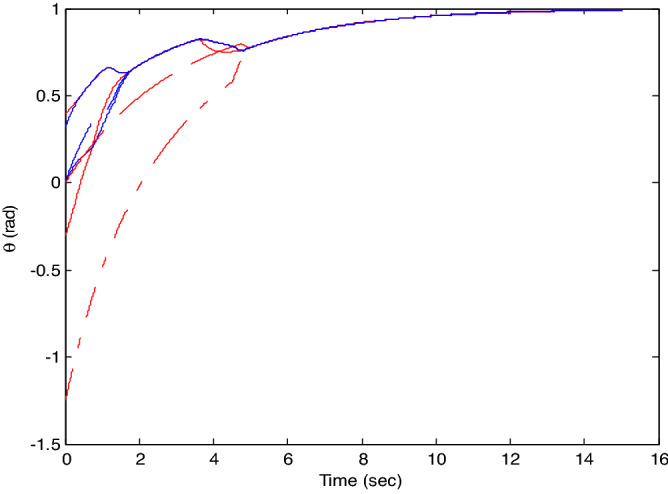
Consensus control on heading angle of seven agents with attacks at one second interval.

**Figure 11:**
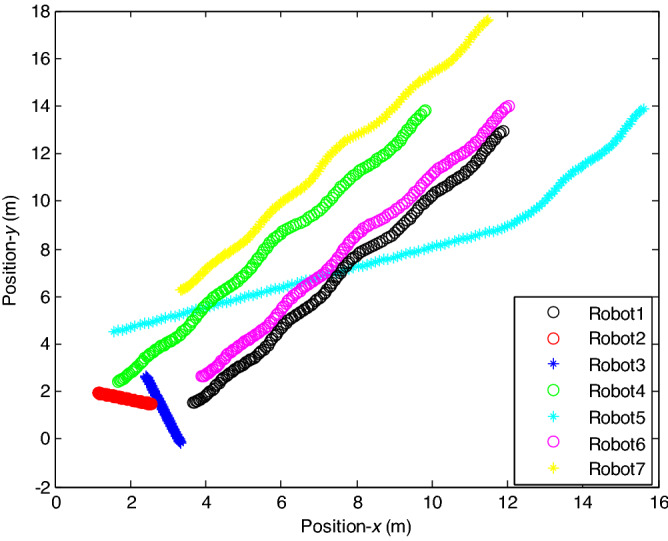
Consensus control of seven agents.

**Figure 12:**
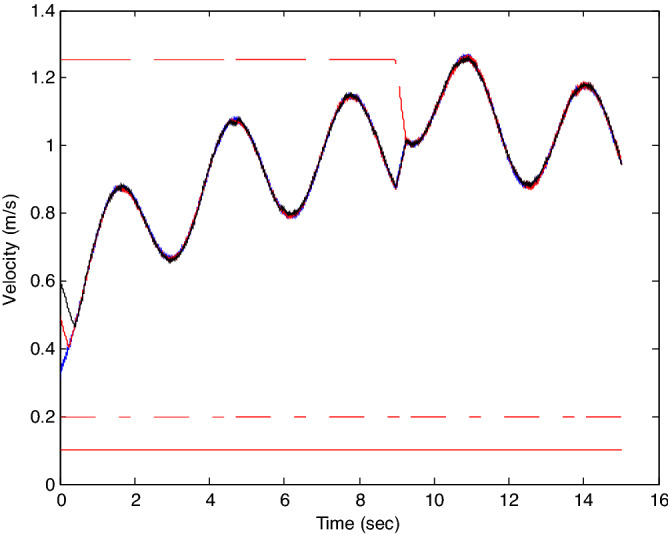
Consensus control on variable velocity of seven agents with attacks.

**Figure 13:**
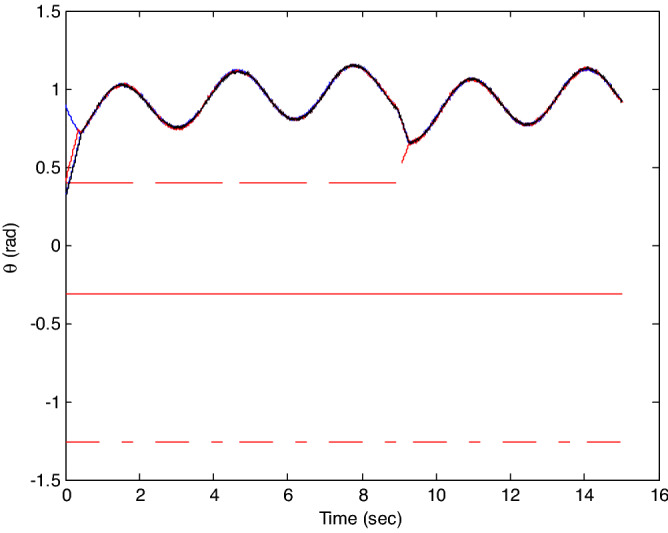
Consensus control on variable heading of seven agents with attacks.

The probability of denial-of-service attack $$\alpha = 0.3$$. The upper bound of continuous attack time $$d_{k} = 4$$ and the initial states of all agents are $${\varvec{x}}_{0} (0) = \left[ {\begin{array}{*{20}c} 2 & {5.1} \\ \end{array} } \right]^{T} ,$$$${\varvec{x}}_{1} (0) = \left[ {\begin{array}{*{20}c} 2 & {4.8} \\ \end{array} } \right]^{T} ,$$$${\varvec{x}}_{2} (0) = \left[ {\begin{array}{*{20}c} {2.5} & 2 \\ \end{array} } \right]^{T} ,$$$${\varvec{x}}_{3} (0) = \left[ {\begin{array}{*{20}c} 3 & 2 \\ \end{array} } \right]^{T} ,$$$${\varvec{x}}_{4} (0) = \left[ {\begin{array}{*{20}c} {2.1} & 2 \\ \end{array} } \right]^{T} ,$$$${\varvec{x}}_{5} (0) = \left[ {\begin{array}{*{20}c} 2 & {4.5} \\ \end{array} } \right]^{T} ,$$$${\varvec{x}}_{6} (0) = \left[ {\begin{array}{*{20}c} {2.5} & {4.6} \\ \end{array} } \right]^{T} .$$ The control time delay $$h_{1} = 3$$, and the output time delay $$h_{2} = 2$$.Note that the matrices $$A_{x}$$ and $$A - K_{i} (k)CA(\forall i \in N)$$ matrices are of Schur stability. Figure 5 shows the motion simulation of the consensus control of agents at constant velocity and heading angle, Fig. 6 shows the velocity consensus control and Fig. 7 shows the heading angle consensus control. The No. 3 Robot agent cannot communicate with the other Robot agents for a time delay due to the network attacks which does not lead to realizing the real-time acquisition of information, leading to straggling. At the initial time No. 2 Robot agent cannot communicate with the other Robot agents for a short delay due to the network attacks, but then No. 2 Robot adopts the states estimator to compensate the time delay at the sixth second, it can follow the other agents’ states, and meanwhile the No. 3 Robot can not follow the other agents’ states, which leads losing its way. Figure 8 shows the motion simulation of the consensus control of agents at constant velocity and heading angle with one second interval attacks. Figure 9 shows the velocity consensus control and Fig. 10 shows the heading angle consensus control. Although the existing interval attacks, the seven agents with estimators can reach consensus on velocity and heading. All the seven agents can realize the real-time consensus acquisition of information. Figure 11 shows the motion simulation of the consensus control of seven agents at variable velocity and heading angle with attacks. Figure 12 shows the velocity consensus control and Fig. 13 shows the heading angle consensus control. At the initial time No. 2, No. 3 and No. 5 Robots cannot communicate with the other Robots for a long time due to the network attacks, but then No. 5 Robot adopts the states estimator to compensate the time delay at the ninth second, it can follow the other agents’ states, and meanwhile the No. 2 and No. 3 Robots can not follow the other agents’ states, which leads losing their way.
